# Mesenchymal Stem Cells in the Pathogenesis and Therapy of Autoimmune and Autoinflammatory Diseases

**DOI:** 10.3390/ijms242216040

**Published:** 2023-11-07

**Authors:** Lina N. Zaripova, Angela Midgley, Stephen E. Christmas, Michael W. Beresford, Clare Pain, Eileen M. Baildam, Rachel A. Oldershaw

**Affiliations:** 1 Institute of Fundamental and Applied Medicine, National Scientific Medical Center, 42 Abylai Khan Avenue, Astana 010000, Kazakhstan; zaripova_lina@list.ru; 2 Department of Musculoskeletal and Ageing Science, Institute of Life Course and Medical Sciences, Faculty of Health and Life Sciences, University of Liverpool, William Henry Duncan Building, 6 West Derby Street, Liverpool L7 8TX, UK; 3 Department of Women and Children’s Health, Institute of Life Course and Medical Sciences, University of Liverpool, Institute in the Park, Alder Hey Children’s NHS Foundation Trust, Liverpool L14 5AB, UK; a.midgley1@salford.ac.uk (A.M.); m.w.beresford@liverpool.ac.uk (M.W.B.); clare.pain@alderhey.nhs.uk (C.P.); 4 Department of Clinical Infection, Microbiology and Immunology, Institute of Infection and Global Health, Faculty of Health and Life Sciences, University of Liverpool, The Ronald Ross Building, 8 West Derby Street, Liverpool L69 7BE, UK; sechris@liverpool.ac.uk; 5 Department of Paediatric Rheumatology, Alder Hey Children’s NHS Foundation Trust, East Prescott Road, Liverpool L14 5AB, UK; 6 Department of Paediatric Rheumatology, The Alexandra Hospital, Mill Lane, Cheadle SK8 2PX, UK; aetab@btinternet.com

**Keywords:** mesenchymal stem cells, immunogenicity, immunomodulation, mesenchymal stem cell dysfunction, mesenchymal stem cell transplantation, autoimmune, autoinflammatory, autologous mesenchymal stem cells, allogeneic stem cells

## Abstract

Mesenchymal stem cells (MSCs) modulate immune responses and maintain self-tolerance. Their trophic activities and regenerative properties make them potential immunosuppressants for treating autoimmune and autoinflammatory diseases. MSCs are drawn to sites of injury and inflammation where they can both reduce inflammation and contribute to tissue regeneration. An increased understanding of the role of MSCs in the development and progression of autoimmune disorders has revealed that MSCs are passive targets in the inflammatory process, becoming impaired by it and exhibiting loss of immunomodulatory activity. MSCs have been considered as potential novel cell therapies for severe autoimmune and autoinflammatory diseases, which at present have only disease modifying rather than curative treatment options. MSCs are emerging as potential therapies for severe autoimmune and autoinflammatory diseases. Clinical application of MSCs in rare cases of severe disease in which other existing treatment modalities have failed, have demonstrated potential use in treating multiple diseases, including rheumatoid arthritis, systemic lupus erythematosus, myocardial infarction, liver cirrhosis, spinal cord injury, multiple sclerosis, and COVID-19 pneumonia. This review explores the biological mechanisms behind the role of MSCs in autoimmune and autoinflammatory diseases. It also covers their immunomodulatory capabilities, potential therapeutic applications, and the challenges and risks associated with MSC therapy.

## 1. Introduction

Mesenchymal stem cells (MSCs) are multipotent progenitor stromal cells that self-renew and differentiate toward multiple mesenchymal cell lineages [1]. With the rapid expansion of research into tissue-specific stem/progenitor populations, in 2006 the International Society for Cellular Therapy (ISCT) defined the minimal criteria for MSC characterisation to include the following: (1) adherence to tissue culture plastic and fibroblastic morphology; (2) positive/negative expression of panels of surface antigens; (3) multi-lineage differentiation toward chondrogenic, adipogenic, and osteogenic cell lineages. The establishment of internationally recognised and standardised criteria for determining what is an MSC population has been fundamental to advancing their role in biomedical research. Identification of MSC phenotype markers and characterisation of their multipotency has led to optimised methods for their isolation and culture from rare populations within tissues. Measurements of phenotype and function provide biological context to tissue-specific differences exhibited between MSC populations and the changes that occur in response to physiological and pathophysiological stimuli. Standardisation of criteria also facilitates the characterisation of MSCs as they undergo bioprocessing protocols in the manufacture of cell-based therapeutics.

**Figure 1:**
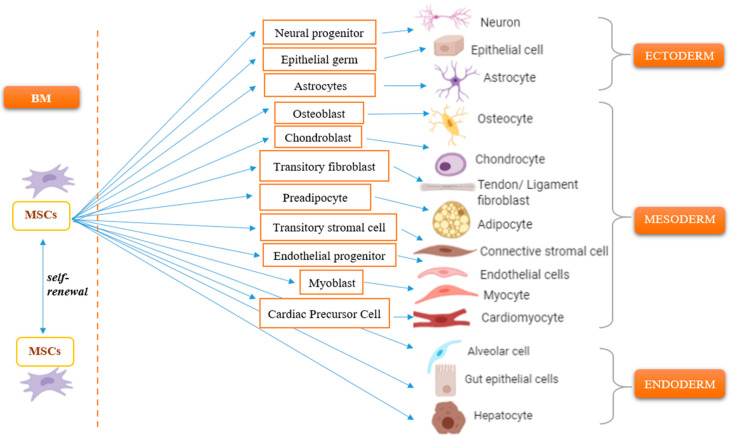
Summary of the mesenchymal stem cell lineage differentiation. MSCs demonstrate multipotent differentiation to cells of mesodermal origin: osteogenic, adipogenic, and chondrogenic pathways. There is some evidence of ectodermal germ (neural, epithelial) and endodermal origin such as alveolar cells, gut epithelial cells, and hepatocytes. Bone marrow, (BM). Created with BioRender.com.

MSCs have been successfully isolated from almost all post-natal mesodermal tissues, including bone marrow (BM), umbilical cord (UC), adipose tissue (AT), amniotic fluid (AF), placenta, dental tissue, synovial membrane, and peripheral blood. Tissue-dependent differences in cell surface antigen expression are indicative of variation in cell migration and cell-homing potential. The reported intra- and inter-tissue functional heterogeneity between MSC clones highlights the need for further understanding of the biology of MSCs and how they can be used effectively in developing cell-based therapeutics [2]. Bone marrow is arguably the most researched tissue source as a result of the seminal work of Friedenstein and colleagues. These studies demonstrated that a sub-population of BM cells, constituting 0.001–0.01% of the total cell number within the tissue [3], was able to undergo osteogenic differentiation and form osseous tissue following heterotrophic transplantation [4,5]. Provided with appropriate stimuli, MSCs have potential for differentiation toward multiple specialised cell lineages of mesenchymal origin, including chondrocytes, osteocytes, tenocytes, ligamentocytes, and myocytes [6]. Differentiation to non-mesodermal cell lineages has been reported with examples of hepatocytes, epithelial cells, alveolar cells, astrocytes, neural precursors, and mature neurons, alluding to the putative role of MSCs in endogenous tissue repair. The understanding of the intrinsic properties of self-renewal and multipotent differentiation is fundamental to their importance in developing advanced regenerative medicine strategies. Specifically, this encompasses the ability to develop and optimise protocols for ex vivo expansion in culture, prior to directed differentiation toward functional cell populations and the manufacture of autologous and allogeneic products that repair and regenerate tissues which have been damaged by injury or disease (Figure 1) [6].

## 2. Migratory Response of Mesenchymal Stem Cells

The migratory response of MSCs is critical to their function. They are recruited in from peripheral blood and home into the site of damaged tissue in response to biochemical cues, where they can moderate inflammatory and immune cell activity, and begin to effect repair [7]. MSC migration and homing to sites of tissue injury is regulated by chemokines, cytokines, and growth factors. It is dependent on the expression of homing receptors and activation of integrins that promote adhesion of MSCs to extracellular matrix proteins. MSCs express a wide range of chemokine receptors including CXCR3, CXCR4, and CCR5 which are involved in the recruitment of MSCs from the bone marrow to the peripheral circulation prior to their migration to the site of injury [8]. The chemokine stromal cell-derived factor-1 (SDF1, known also as CXCL12) is critical for stem/progenitor and mesenchymal cell chemotaxis and organ-specific homing in injured tissue through interaction with its cognate receptor CXCR4 on the surface of these cells [9]. CXCR4 is highly expressed by freshly isolated BM-MSCs from young adults, but becomes reduced with the ageing of endogenous tissues and in vitro ageing as the cells are repeatedly passaged in culture. This therefore limits their ability to respond to homing signals and hence reduces their regenerative capability [8]. Senescence of MSCs has significant consequences on the biology of MSCs, including their self-renewal and proliferative capacity, as well as effector functions, including immunomodulation and cell lineage differentiation and specialisation. CXCR4 gene deletion in young-donor MSCs was associated with the increased production of reactive oxygen species (ROS) and subsequent DNA damage and replicative senescence, which is characteristic of prematurely aged phenotypes [10]. Furthermore, the reduction of CXCR4 on the BM-MSC of aged mice has been shown to be causal in the impaired ability of MSC to support haematopoietic stem cell biology [10].

In response to injury or ischaemia, homing receptor expression and chemokine production is upregulated to stimulate granulocyte colony stimulating factor (G-CSF)-mediated activation and mobilisation of MSCs from BM into peripheral blood. Monocyte chemotactic protein-1 (MCP1) recruits MSCs during the inflammatory response, in contrast to macrophage migration inhibitory factor (MIF) which reduces MSC migration [11].

Bioactive molecules play an important role in immune homeostasis (Table 1). Growth factors, such as basic fibroblast growth factor-2 (FGF2), vascular endothelial growth factor (VEGF), hepatocyte growth factor (HGF), insulin-like growth factor-1 (IGF1), platelet-derived growth factor (PDGF), and transforming growth factor-β1 (TGFβ1), play a prominent role in regulating MSC migration. FGF2 promotes upregulation of αVβ3 integrin and activation of MEK/ERK pathways that stimulate the migration of BM-MSCs and homing to sites of injured tissue [9]. VEGF regulates BM-MSC migration and proliferation through platelet-derived growth factor receptors (PDGFRs) and SDF-1α expression. PDGF has been shown as a prominent factor for BM-MSC migration, binding to PDGFRα and PDGFRβ [9]. Production of TGFβ1 is increased at the site of tissue damage where it stimulates expression of CXCR4 on BM-MSCs and promotes their migration and their homing to myocardial injury [12]. This is most likely by activation of the TGFβ type I receptor and downstream non-canonical signalling by Akt, extracellular signal-regulated kinase 1/2 (ERK1/2), focal adhesion kinase (FAK), and p38 [13].

Another molecule responsible for MSC migration is osteopontin (OPN), which has been reported to be upregulated in response to tissue damage and subsequent inflammation in the heart, bone, kidney, and lung [9]. OPN promotes BM-MSC migration through the increased expression of integrin β1 and lamin A/C expression, leading to a decrease in nuclear stiffness via the FAK-ERK1/2 signalling pathway [67].

Migration of MSCs is also stimulated by pro-inflammatory cytokines, including interleukin-1β (IL1β), tumour necrosis factor-α (TNFα), and interferon-γ (IFNγ) [68]. TNFα is involved in tumour progression and plays an essential role in epithelial-mesenchymal transition [69]. TNFα and IFNγ act in synergy to induce the production of superoxide anions, with corresponding up-regulation of inflammatory responses. IL1β cytokine activates mast cells and induces histamine production, which increases membrane permeability [69]. IL1β was found to promote the expression of CXCR3 on the surface of MSCs through activation of the p38 MAPK signalling pathway [68]. At the same time, IL1β upregulated CXCL9 (both at the mRNA transcript level and measured ligand secretion) in umbilical vein endothelial cells, and this was concurrent with an increase in the chemotaxis and trans-endothelial migration potential of MSCs [68]. However, pro-inflammatory cytokines could play a dual role in MSC migration and immunomodulatory function. Low level expression of pro-inflammatory cytokines has been reported to promote MSC immunomodulation of the inflammatory environment, but at higher concentrations of pro-inflammatory cytokines, such as those present in autoimmune diseases, they have a detrimental impact on MSC biology, leading to impaired function. Detailed research has revealed that pro-inflammatory cytokines, specifically IFNγ and TNFα, synergistically impair proliferation and differentiation of MSCs via nuclear factor kappa-light-chain-enhancer of activated B cells (NFκB) in an experimental murine model [70]. Based on previous research, the concentration and the length of exposure to these cytokines can influence the biological response of MSCs and therapeutic ability by activating MSCs, or inducing MSC death through apoptosis, necroptosis, or autosis [71].

## 3. Immunomodulatory Properties of MSCs

The immunomodulatory ability of MSCs is of significant interest within the context of understanding the underpinning scientific mechanisms that contribute to the dysregulation of immune homeostasis and the causal relationship to the onset and progression of autoimmune and autoinflammatory disorders. Advancing this understanding will have a significant impact on the development and production of therapeutic interventions. MSCs regulate both innate and adaptive immune responses through cell–cell contact and production of paracrine mediators (Table 2). The immunomodulatory mechanisms of MSCs have been studied using in vitro and in vivo experimental animal models of autoimmune disorders [72,73,74].

### 3.1. Paracrine Activity of MSCs

Paracrine activity of MSCs includes the secretion of growth factors and cytokines that regulate immune cell biology, promote angiogenesis, and suppress fibrotic remodelling. Predominant growth factors involved in these processes include VEGF and FGF2, which have both been reported to promote myocardial recovery and improve cardiac function by mediation of angiogenesis and induction of neovascularisation following ischemic injury [75].

Production of IGF1 and TGFβ regulates the MSC-mediated suppression of CD8^+^ T-cells, while HGF and FGF2 suppress fibrotic remodelling [75]. BM-MSCs contribute to lymphopoiesis and regulate the development of T- and B-lymphocytes through the secretion of these growth factors and cytokines, as well as the expression of cell adhesion molecules. HGF and macrophage colony stimulating factor (M-CSF) regulate MSC modulation of dendritic cells (DCs) by inducing differentiation of mature DCs into tolerogenic dendritic cells (DCregs) via the AKT signalling pathway [76]. MCP1 stimulates the activity of regulatory T-cells (Treg), a sub-population of T-cells that regulates immune responses and reduces the onset and progression of autoimmune disease.

MSC-mediated immunosuppression is dependent on IFNγ activation in combination with TNFα or IL1β [77,78]. This phenomenon has been coined the term “licensing” and may offer a mechanism for a role of MSC dysfunction in the activity and remission of autoimmune and autoinflammatory disease states [77]. On stimulation with a combination of IFNγ with TNFα or IL1β, MSCs produce nitric oxide (NO), a powerful cytotoxic molecule that inhibits T-cell proliferation [79,80]. Prostaglandin E2 (PGE2) programs macrophages to release IL10 and inhibit T-helper cell activity and IL2 production. Inhibition of this prostaglandin has been shown to result in a decrease in the anti-proliferative effect exhibited by MSCs on T-cells. Another soluble mediator that contributes to MSC-mediated immunosuppression is indoleamine 2,3-dioxygenase (IDO), an enzyme that catabolises the essential amino acid tryptophan in the kynurenine pathway [81]. IDO released by MSCs in response to IFNγ reduces tryptophan availability and the production of metabolite derivatives in NK cells and T-cells and therefore inhibits their proliferation [81]. In addition, MSCs secrete immunosuppressive cytokines, including IL7, IL11, IL14, and IL15, and stimulate an increase in anti-inflammatory cytokine IL10 production by DCs and monocytes [82].

#### Extracellular Vesicles Derived from MSCs

More recent investigation has been directed to the secretion of paracrine immunomodulatory factors, which are packaged into extracellular vesicles (EVs) to form the bioactive fraction of the MSC secretome [83]. This has elucidated the mechanisms by which the MSC secretome mediates its effector functions and has provided multiple examples of the potential therapeutic properties of the EVs [84]. EVs are heterogeneous structures that can be subtyped to exosomes, microvesicles, and apoptotic bodies. Exosomes are created by an endosomal route and are typically 30 to 150 nm in diameter. They are derived when MSCs exchange genetic material between cells, particularly microRNA and mRNA. EVs contain bioactive cytoplasmic and membrane proteins, including tetraspanins (CD81, CD63, and CD9), heat-shock proteins (HSP60, HSP70, and HSP90), ALIX, tumour susceptibility gene 101 (TSG101), enzymes, and extracellular matrix proteins [85]. MSC-derived exosomes can be transferred between cells with microRNA cargo enabling the regulation of cell cycle and migration (miR-191, miR-222, miR-21), inflammation (miR-204-5p, miR-181c), and angiogenesis (miR-222 and miR-21) [85]. Examples of the ability of MSC-derived exosomes to induce T-regulatory cells (T-regs) have been demonstrated in vitro, with MSC-derived exosomes showing increased polarisation of naïve T-helpers to CD4^+^CD25^+^Foxp3^+^ Treg cells in the presence of allogeneic antigen-presenting cells [86]. MSC-derived exosomes were investigated in an in vivo mouse model of graft-versus-host disease (GVHD). Mice were irradiated with 100 cGy and then treated with 1–2 × 10^7^ human peripheral blood mononuclear cells (PBMCs) injected via a tail vein and MSC-derived exosomes injected intraperitoneally [86]. MSC-derived exosomes decreased the combined disease activity score, including weight loss, activity, posture, skin, and hair integrity, and improved the percentage of animals surviving to the study end-point on day 34 (*p* < 0.05) [86]. The positive effect of MSC-derived exosomes on the survival of mice in the GVHD model has been explained in another study where it was shown that MS-EVs induced Treg-associated effects on anti-CD3/CD28-stimulated PBMCs [87]. All together, these studies demonstrated that human MSC exosomes could induce both human and mouse Tregs from APC-activated T cells, providing a potential opportunity for human application.

MVs are membrane vesicles that differ from other EVs by their size, ranging between 100 nm up to 1 μm in diameter, and in density of 1.04–1.07 g/mL [88]. Microvesicles (MVs) are formed by outward budding or pinching of the MSC plasma membrane and contain cytosolic and plasma membrane-associated proteins, cytoskeletal proteins, heat shock proteins, integrins, and RNA molecules, such as mRNA, miRNA, snoRNA, and rRNA [89,90]. Western blotting of MSC-derived MVs showed the presence of CD9, CD63, CD81, TSG101, tryptophanyl-TRNA synthase 1, C1q, and calnexin. In contrast, MSC-derived exosomes were characterised by positivity to CD63, CD81, and TSG101, and negativity to calnexin [88,90]. The mechanism of MV release from MSCs and their function also differs from other types of extracellular vesicles. MVs play a critical role in regulating paracrine/endocrine factor-mediated signalling between MSCs and differentiated specialised cells [91]. They are derived from cells through outward budding dependent on mitochondria-mediated calcium signalling. MVs that are released from damaged cells will deliver specific cargo to instruct naïve MSCs to become immunomodulatory or trigger their differentiation to repair tissues [91]. In response, MSC-derived MVs home into the sites of tissue inflammation to deliver proteins/peptides, mRNA, microRNA, lipids, and/or organelles with reparative and anti-inflammatory properties [92].

MVs mediate cell–cell communication by contact with specific ligands on relevant cell types and transfer their cargos (membrane proteins or different types of RNAs) from MSCs to other cells, and therefore may be useful in therapeutic applications [90]. For instance, MVs have been used for the transport of small therapeutic components, such as the delivery of paclitaxel to pancreatic cancer cells to reduce proliferative activity of cells [84].

The anti-inflammatory effect of MSC-derived MVs was tested in a human model of bacterial pneumonia, where *E. coli* were instilled intra-bronchially in human donor lungs not used for transplantation [93]. One hour later, 200 microlitres of MVs purified from 20 million MSCs were administered into the perfusate as therapy. At six hours post-administration, the MV-treated lung showed increased alveolar fluid clearance by 144% compared with the control lung lobe and significantly reduced lung protein permeability as measured using Evans Blue dye. After treatment with MSC-derived MVs, the level of TNFα in bronchoalveolar lavage fluid was reduced by 72% and the bacterial count in the injured alveolus decreased (though not statistically significant in the study) [93].

The administration of human Wharton–Jelly MSC-derived MVs in a renal transplantation rat model was shown to improve renal function and survival of the rats [94]. The administration of MVs after renal transplantation led to a 39.13% lower expression of von Willebrand Factor (a marker of endothelial injury). Pro-inflammatory TNFα was 65.7% lower in serum, whereas anti-inflammatory IL10 levels were 25.19% higher when compared to the control group without MSC-derived MVs [94]. At two weeks post-transplantation, co-delivery with MVs was shown to reduce apoptosis of renal cells, significantly reduce fibrotic lesions, as identified by Masson’s trichrome staining, and decrease CD68^+^ macrophage infiltration in the kidney [94].

MSC-derived exosomes and MVs have been extensively studied, however, there is limited information on the function of apoptotic bodies (ABs) [95]. ABs are released after apoptosis of cells as the plasma membrane separates from the cytoskeleton. They are the largest type of extracellular vesicle (1–5 mm in diameter) containing intracellular fragments, mitochondria, Golgi apparatus, and endoplasmic reticulum [89]. ABs facilitate intercellular communication and are key mediators in processes that include tissue homeostasis, pathogen dissemination, and immunity [95]. It has been demonstrated in vivo and in vitro that ABs target macrophage populations, promoting their polarisation towards the anti-inflammatory M2 phenotype with increased secretion of IL10 and TGFβ [96]. Transplantation of ABs in murine skin wound healing models demonstrated macrophage polarisation towards an anti-inflammatory M2 phenotype followed by significantly enhanced cutaneous wound healing [96]. ABs derived from MSCs have not demonstrated a direct effect on fibroblasts, however, conditioned medium from macrophages treated with ABs enhanced the migration and proliferation of fibroblasts in scratch wound assays and by Ki67 immunofluorescence staining [96].

Together, EVs derived from MSCs demonstrate immunomodulatory functions in vitro [90,97]. MSC-secreted EVs influence immune cells, including impairment of DC maturation as exhibited by reduced expression of CD83, CD38, and CD80, increased production of TGFβ, and decreased secretion of IL6 and IL12p70 [97]. MVs derived from MSCs treated with IFNγ have been shown to increase CD4^+^CD25^+^FoxP3^+^ Treg populations in the presence of TGFβ1 in vitro, however, native MVs are less effective in inducing Tregs [90].

Immunoregulatory and regenerative properties of MSCs are also mediated by the transfer of mitochondria. Mechanisms of mitochondrial trafficking have been proposed to include tunnelling nanotubes, gap junctions, extracellular vesicles, and cell fusion. The mechanism for the transfer of mitochondria within tunnelling nanotubes is mediated by motor-adaptor protein complexes related to the mitochondrial Rho GTPase, Miro1. Miro1 is important for transferring mitochondria between cells. MSCs overexpressing Miro1 have been shown to have enhanced rescue of epithelial cells by reducing airway hyper-responsiveness and production of pro-inflammatory cytokines, and enabling the restoration of physiologic ATP production [98]. Another mechanism for the transport of mitochondria between cells is by gap-junction communication mediated by the transmembrane protein Connexin-43. Connexin-43 forms hemichannels in association with other connexin proteins to allow for direct exchange of metabolites and microRNAs [98,99].

Microtubule and gap junction-mediated transfer of mitochondria from MSCs to damaged immune cells, cardiomyocytes, neurons, renal tubular cells, and alveolar and bronchial epithelial cells has been widely investigated [98,99]. Mitochondrial transfer between MSCs and other somatic cells is initiated in pathophysiological environments and is predominantly triggered by damaged mitochondria or mitochondrial DNA and the accompanying elevated production of ROS, which are released from ruptured cells [100]. Additionally, MSCs transfer mitochondrial DNA (mtDNA) to other cells by extracellular vesicles (EVs) and cell fusion by rearrangement of the actin cytoskeleton and fusogenic glycoproteins across the membranes [98]. Mitochondria labelled with an MT-specific fluorescent probe (MitoTracker Green) showed that the transfer of mitochondria from MSCs to human PBMCs mainly engaged T-helper CD4^+^ lymphocytes, rather than T-cytotoxic CD8^+^ lymphocytes. Transcriptomic RNA sequencing showed T-cell activation (IL2RA-CD25) and differentiation to T-regs (CD4^+^CD25^+^Foxp3^+^) with the upregulation of FoxP3, CTLA4, and GITR mRNA levels validated by qPCR [101].

Furthermore, in a murine model of GVHD, the transfer of mitochondria from MSCs to PBMCs resulted in a significant decrease in Th1 (CD4^+^IFN-γ^+^) and cytotoxic T-cell (CD8^+^IFN-γ^+^) infiltration (*p* < 0.003), while T-reg cells were slightly elevated [101]. This led to a significant improvement in tissue damage in the spleen, small intestine, liver, and lung [101]. Thus, the mice receiving mitocepted PBMCs had a 34.7% reduction in liver pathology scores, a 57.04% decrease in lung damage scores, and a 25.35% reduction in small intestine problem scores based on crypt regeneration and loss of enterocyte ulceration [101]. Reduction of tissue injury was accompanied by a 27% improvement in the mouse survival rate compared with controls [101].

### 3.2. Regulatory Effects of MSCs on Immune Cells

Contact-dependent mechanisms of MSC-mediated immunosuppressive activity inhibit the proliferation and activation of the major immune cell populations, including T-lymphocytes, B-lymphocytes, DCs, pro-inflammatory macrophages, and natural killer (NK) cells by arrest in the G0/G1 phase of the cell cycle [102]. Cell–cell interactions between MSCs and immune cells are mediated by adhesion molecules, including P-selectin, intercellular adhesion molecule-1 (ICAM1), and vascular cell-adhesion molecule-1 (VCAM1, CD106). It was found that chemokines and adhesion molecules trigger T-cells rolling, arrest, and then transmigration through the endothelium. An inflammatory environment induces MSCs to secrete multiple chemokines and upregulate the expression of ICAM1 and VCAM1, which attract and engage T-cells to MSCs [79]. The clinical relevance of these interactions is highlighted by showing that the blockade or deletion of ICAM1 and VCAM1 could significantly reverse MSC-mediated immunosuppression in vitro and in vivo [103]. Moreover, high expression of ICAM1 and VCAM1 is associated with greater immunosuppressive capacity of MSCs [103]. MSCs inhibit the proliferation of T-cells, specifically pro-inflammatory populations of T-helper cells (Th17 and Th1), decrease the ratio of Th1/Th2 T-helper cell populations, and promote an anti-inflammatory profile by activation of Treg cells [77]. These findings could be translated into therapies for autoimmune and autoinflammatory diseases such as rheumatoid arthritis (RA), which are characterised by a predominance of pro-inflammatory CD4^+^ T cells with the hyper-proliferative capacity to differentiate into Th1 and Th17 pathogenic T cells [104]. Th17 cells participate in the pathogenesis of different autoimmune diseases, such as systemic lupus erythematosus, type 1 diabetes, multiple sclerosis, and bowel disease [105]. Moreover, MSCs have been shown to be highly stimulatory to Treg populations in both in vitro and in vivo studies [106,107]. In a murine model of autoimmune encephalomyelitis, MSCs increased demethylation of the Treg-specific demethylated region (TSDR) and upregulated the expression of Runx complex genes of Foxp3 (Runx1, Runx3, and CBFB) in the TSDR [107]. The induction of Tregs by MSCs has been considered to be caused by direct cell–cell contact as well as the secretion of PGE2, TGFβ1, IL10, and soluble human leukocyte antigen-G (sHLA-G) [106,107]. The balance between Treg cells and Th17 cells determines the efficacy of immune therapy and thus underscores the importance of MSCs as tools for moderating autoimmune and autoinflammatory diseases.

Another mechanism of MSC modulation of T-cell activity is the impairment of leukocyte migratory potential by inhibition of the adhesion molecules and receptors on the cell surface of T-cells and the endothelial cell membrane [72]. For instance, MSCs reduce the level of ICAM1, α4, and β2 integrins, as well as CXCR3 expression, regulating T-cell trafficking across the endothelial blood–brain barrier [72].

Additional evidence has shown that MSCs can inhibit the differentiation, maturation, and activation of DCs [97]. DCs are highly specialised antigen-presenting cells that play an exclusive role in naïve T-cell stimulation during the primary immune response. MSCs inhibit the initial differentiation of monocytes to DCs by dampening the expression of CD86, CD1a, and HLA-DR. Furthermore treatment of DCs with MSC-derived EVs demonstrated a reduced ability to migrate toward the CCR7-ligand CCL21 [97]. MSCs significantly influence DC antigen presentation to CD4^+^ T-cells and cross-presentation to CD8^+^ T-cells because of the inability of DCs to migrate to the draining lymph nodes [108]. The influence of MSCs on B-cells has been less well studied, although it is known that the interaction between MSCs and B-cells is complex with the interplay of multiple different contributing factors. MSCs can regulate B-cell activation indirectly through T-helper cell activity or directly through the production of soluble factors, including the IL1 receptor antagonist. Luk et al. (2017) demonstrated that adipose tissue-derived MSCs treated with 50 ng/mL IFNγ for 96 h were able to significantly reduce B-cell proliferation and inhibit B-cell IgG production. MSCs are able to reduce plasmablast formation and promote the induction of regulatory B-cells (Bregs) and IL10 production [102]. In the presence of T-cells, MSCs also inhibit the proliferation of B-cells, which could be mediated by T-cell-secreted IFNγ, since MSCs pre-treated in vitro with exogenous IFN-γ are able to inhibit B-cell proliferation [102]. Thus, MSCs can negatively influence abnormal proliferation and autoantibody production by B-cells, providing a mechanistic basis that has significant implications for the development of autoimmune disease therapies, such as rheumatoid arthritis (RA) and systemic lupus erythematosus (SLE). The proliferation of B-cells results in the release of autoantibodies in the form of IgM and/or IgG, rheumatoid factors in RA, or antinuclear antibodies in SLE. MSCs have been shown to induce regulatory immune cells and suppress T-helper and B-cell responses, reducing both IgM and IgG production in mouse models and patients with lupus nephritis [109]. Cell-mediated interactions between MSCs and NK cells may impact on the immunobiology of both cell types. NK cells can lyse pathogen-infected or transformed target cells without the aid of prior immunisation, or can be activated by IL2, IL12, IL15, IL18, IL21, IFNα, and IFNβ [110]. MSCs are able to suppress the proliferation of NK cells and stimulate their degranulation, but at the same time MSCs promote NK production of IFNγ and TNFα [111]. Conversely, NK cells activated with IL2, IL12, and IL15 have been shown to release IFNγ, TNFα, perforin, and granzymes, and mediate MSC lysis [40]. Naïve NK activation has also been shown to increase production of ROS leading to decreased BM-MSC viability [111]. The complexity of NK interactions with MSCs, their function in maintaining immune cell homeostasis, and the pathophysiologic complications on dysregulation that leads to the progression of autoimmune and autoinflammatory disorders are more comprehensively discussed in the review by Jewett et al. (2012) [112].

## 4. Immunogenicity of MSCs

MSCs are considered immune-privileged, having low expression of major histocompatibility complex (MHC) class I, minimal expression of MHC class II, and deficiency in co-stimulatory molecules required for immune cell activation, including B7-1, B7-2, or CD40 [81].

Contrary evidence suggests that MSCs can also be immunogenic. Animal studies have revealed that despite low level immunogenicity, allogeneic MSCs are immune-rejected via MHC ClassI and MHC ClassII in mice [113]. Oliveira et al. (2017) suggested that rejection of MSCs might be dependent on the context of the inflammatory environment into which the cell population was transplanted. The study showed that prior treatment of MSCs by IFNγ and TNFα could modulate MHC class I and II expression, increasing their immunogenic potential [81]. This immune recognition of MSCs has been proposed as an important mechanism in attaining an immunomodulatory therapeutic effect. Witte et al. (2018) showed that allogeneic Umbilical Cord (UC)-MSCs were recognised by host immune cells and phagocytosed by monocytes post-infusion into mice. The subsequent UC-MSCs-primed monocytes demonstrated an increase in IL10 and TGFβ gene expression and reduced TNFα expression; moreover, monocytes primed by UC-MSCs have been shown to induce Treg cell differentiation in mixed lymphocyte reactions [114]. However, prolonged treatment of MSCs with pro-inflammatory cytokines IFNγ, TNFα, IL17, and IL1β resulted in not only activation but also increased expression of MHC class I/II [115]. Considering potential clinical applications of MSC delivery into the inflammatory tissue, this may influence the balance between immunosuppressive activity and MHC Class II expression by MSCs [116].The safety concerns of MSCs transplantation have also included the potential of the risk of thrombosis. Intravascular transplantation of tissue factor (TF)-bearing cells provokes an instant blood-mediated inflammatory reaction (IBMIR) resulting in thrombotic complications and reduced engraftment [117]. Plasma levels of TF/CD142 are correlated with activation of the IBMIR and vary between MSC from different sources [117]. AT- and UC-MSCs demonstrate higher levels of TF, reduced hemocompatibility, and increased clot formation dependent on coagulation factor VII [117]. MSCs highly express pro-thrombotic tissue factor (TF/CD142) and collagen type-1, which activate the coagulation cascade [118]. The tissue factor (TF)-mediated pro-coagulant activity could be reverted by heparin co-administration in MSC transplantation. In addition, investigation of the hemocompatibility of AT-, UC-, and BM-MSCs revealed that reducing the TF/CD142^+^ subpopulation significantly improved hemocompatibility of MSCs and consequently decreased the risk of thrombosis [117]. Mitigation of these safety concerns will need to include robust pre-clinical and clinical trial investigation. Bio-processing protocols directing the isolation, culture, and manufacture of MSC-based therapies need to be tightly regulated with appropriate quality control (QC) assays defined to evaluate the phenotype and biological function of MSCs as they form the final therapeutic product.

Long-term ex vivo expansion in the production of MSC therapies has been reported to increase pro-thrombotic properties. Infusion of large cell doses of higher passage MSCs (passages 5–8) have been shown to elevate the coagulation cascade, cause activation of complement marker C3a, and increase the expression of thrombin, FVII, FXIa, and FXIIa clotting factors that may cause thrombosis or embolism [118]. This highlights the need for hemocompatibility assessment of MSC products before intravascular delivery.

Together, these studies have shown that MSCs are not absolutely immune-privileged. At the same time, it is recognised that local immune suppression, for example with anti-CD45 immunotherapy or cyclosporine A, could mask MSC immunogenicity [81]. Nevertheless, Thompson et al. (2020) reviewing 55 randomised controlled trials of MSC therapy, including 2696 patients, concluded that MSC transplantation was associated with an increased risk of fever compared to controls while other side effects of treatment, such as post-infusion infection, thrombosis, or malignancy were not recorded [119].

## 5. Impairment of MSC Biology as a Key Moment in Disease Pathogenesis

There is now an increased understanding of the role of MSCs in the mechanisms of development and progression of autoinflammatory and autoimmune diseases. MSCs respond to tissue damage by reducing inflammation and repairing injured tissue as a normal physiological response. In pathophysiological autoimmune and autoinflammatory conditions, which are characterised by consistent chronic inflammation, MSCs are passive targets in the inflammatory process. They become impaired and exhibit loss of immune modulatory function. Impairment of MSC biology has been identified in RA, ankylosing spondylitis (AS), systemic lupus erythematosus (SLE), systemic sclerosis (SSc), chronic obstructive pulmonary disease (COPD), Parkinson’s disease, type 2 diabetes, and idiopathic pulmonary fibrosis (IPF). MSC impairment is manifest by a reduction in proliferative capacity and immunoregulatory properties, altered morphology, dysregulated cytokine secretion, and altered cell–cycle regulation with enhanced senescence and reduced capability in supporting the hematopoietic system [120,121,122,123,124,125] (Table 3).

MSCs are negatively influenced by the high concentrations of pro-inflammatory cytokines that are present within the pathogenic environment of autoimmune and autoinflammatory diseases [54,55]. Pro-inflammatory cytokines, specifically IFNγ and TNFα, synergistically impair proliferation and differentiation of MSCs via NFκB [70,128]. It has been shown in previous research that treatment with high levels of IFNγ and TNFα for a period of 21 days resulted in NFκB–mediated upregulation of the oncogenes c-Fos and c-Myc, followed by increased susceptibility to MSCs in tumorigenesis. Medications that reduce the levels of IFNγ and TNFα (e.g., aspirin) block malignant transformation of MSCs by inhibition of NFκB/SMAD7 and NFκB/c-FOS and c-MYC pathways in mice [70,128]. These findings suggest that autoimmune disorders are associated with suppressed MSC function and the induction of MSC tumorigenesis by NFκB–mediated oncogene activation [70,128]. These findings have further implications for the clinical application of MSCs if they are to be delivered into the pro-inflammatory environment present within autoimmune and autoinflammatory diseases, with robust evaluation of clinical trial evidence required to measure the safety and efficacy of the therapeutic applications.

Interestingly, BM-MSCs treated with TNFα and TGFβ1 elevate gene expression of pro-inflammatory mediators CCL2 and CXCL8 through the NFκB/p65 pathway and COX2 through SMAD3 activation [55]. This data highlights the importance of the microenvironment in regulating the pro-inflammatory fate of MSC function.

Moreover, MSCs stimulated by TNFα and IL1β for up to 18 days obtained what was described as a cancer-associated fibroblast (CAFs) morphology, inclusive of increased cell size, detected by calcein and Hoechst staining, accompanied by elevated levels of vimentin and fibroblast activation protein (FAP), and reduced expression of α-smooth muscle actin (αSMA). These cells were characterised by the release of pro-inflammatory factors and stimulated cancer cell migration by CCR2, CCR5, CXCR1/2, and Ras-activating receptors and therefore may be considered as pro-carcinogenic [129].

Another crucial mechanism in the impairment of MSC function is highlighted in RA by the reduced ability to downregulate Th17 cell activity [122]. RA-derived MSCs have lower proliferative potential and migration capacity, which does not correlate with previous treatment with methotrexate or biological agents, including TNFα inhibitors and anti-IL1. Additionally, the chondrogenic potential of synovial MSCs was impaired in direct relation to synovial inflammation measured using the arthroscopic visual analogue score in RA patients [126]. MSCs isolated from patients with active RA have been shown to be defective in their ability to support haematopoiesis. Abnormalities of both BM-derived haemopoietic cells and MSCs are indicative of impairment in the immunosuppressive and haematopoiesis-supporting functions of MSCs, which could contribute to the initiation and progression of disease [130].

MSCs isolated from AS patients showed normal rates of proliferation, cell viability, expression of cell surface CD antigens, and potential for multi-lineage differentiation. However, their immunomodulatory properties measured in two-way mixed-lymphocyte reaction (MLR) or PBMC proliferation in the presence of phytohemagglutinin were weaker compared to MSCs from healthy volunteers [123]. MSCs obtained from AS patients have decreased phosphorylation of Beclin-1, an important molecule required for the initiation of autophagy, resulting in the deficiency of autophagy, and as a consequence MSC dysfunction [131]. Autophagy is a lysosome-mediated catabolic process that eliminates molecules and cellular components, including nucleic acids, proteins, and lipids [132]. Autophagy participates in many physiological and pathological processes and can be affected by pro-inflammatory mediators such as lipopolysaccharide (LPS). Li et al. (2017) demonstrated that the basal level of autophagy was equal in MSCs from healthy donors and AS patients, however, LPS-induced autophagy was weaker in AS-MSCs than in healthy MSCs [131]. The level of autophagy reflects the physiological/pathophysiological status of MSCs and abnormal autophagy is included in the pathogenesis of many autoimmune diseases, including inflammation in AS [131].

BM-MSCs derived from patients with SLE show impaired immunomodulatory properties and reduced proliferation rates. This phenomenon was coupled with increased ROS production, DNA damage, expression of senescent p16 and p53, altered cytokine profile with overexpression of pro-inflammatory IL6 and IL8, and downregulation of TGFβ1, IDO, and LIF [127]. SLE BM-MSCs that have been chronically stimulated by pro-inflammatory cytokines within the native tissue environment exhibit a pathophysiological and senescent phenotype with over production of pro-inflammatory mediators that promote inflammation and cellular dysfunction [127]. It was shown that SLE MSCs have a five-fold increase in IFNβ and increased IFNβ-induced mRNAs, including mRNA for the intracellular nucleic acid sensing adaptor protein MAVS. Lin et al. (2017) proposed that the IFNβ-MAVS feedback loop may alter the development of immune cells and contribute to autoimmune progression in SLE [127]. Alterations in MSC function in SLE may affect the bone marrow stromal microenvironment that regulates haematopoiesis, contributing to altered immune responses. In systemic sclerosis (SSc) where the main feature of pathogenesis is vascular damage, there is impaired differentiation of MSCs toward the endothelial cell lineage [124]. Human MSCs and endothelial cells express vascular endothelial growth factor receptor-1 (VEGFR1), VEGFR2, and vascular cell adhesion molecule-1 (VCAM1). SSc derived-MSCs were characterised by early senescence, reduced migration, and antigenic potential, and have been predicted to affect endothelial repair following chronic ischemia in this disease [124].

## 6. Pre-Clinical Studies of Mesenchymal Stem Cells

The combined properties of immunomodulation and differentiation, hematopoietic support, and pro-regenerative features account for the promising therapeutic potential of MSCs. Particular attention is given to their potential efficacy in cases of severe autoimmune or autoinflammatory diseases that are refractory to conventional therapy, and the opportunity for fewer side effects when compared to the need for repeated administration of immunosuppressive drugs. Recent pre-clinical studies focused on stem cell therapy have demonstrated the efficacy and safety of MSC transplantation [133,134,135].

MSC transplantation was proposed as a promising new direction for chronic lung disease. Pre-clinical investigations revealed the efficacy of intratracheally, intranasally, or systemically administered MSCs obtained from BM, AT, UC, or placenta in lung injury models [136]. MSCs are localised to the lung after systemic administration by their ability to home into the sites of injury through the engagement of chemotactic proteins, such as SDF1/CXCL12 with CXCR4. In injured lung animal models, MSCs regenerated lung tissue, reduced inflammation, and limited fibrosis by upregulating anti-inflammatory and downregulating pro-inflammatory cytokine release [136]. MSCs localised to the lung following bleomycin-induced injury in mice arrested the progression of fibrosis and decreased inflammation [136]. Studies using MSCs in experimental murine models of asthma identified immunosuppressive effects of MSC by recruitment of CCR2^+^ monocytes and increased IL10 production [137]. The immune suppressive effects of MSC in the model of asthma also included elevated levels of TGFβ, transfer of mitochondria to airway epithelial cells, and increased numbers of Tregs [137]. However, MSCs display a dual role in the progress of fibrosis. Despite the immunomodulatory and anti-inflammatory properties of MSCs, TGFβ is a primary factor in driving fibrosis via activation of Smad-based and non-Smad-based signalling pathways. This results in activation of myofibroblasts, enhanced production of extracellular matrix (ECM), and inhibition of its degradation [138]. Further in-depth studies examined the dual role of TGFβ as an anti-inflammatory mediator during the acute phase of injury but determined that investigation of the long-term effects of pro-fibrotic TGFβ production are needed to explore the safety of MSC therapy, including their optimal dosage and route of administration. The immunomodulatory and regenerative properties of UC-MSCs have been demonstrated following intraperitoneal transplantation into a rat model of collagen-induced arthritis (CIA) [73,139]. The administration of UC-MSCs at a dose of 2 million cells per rat showed significant improvement in the reduction of joint inflammation and general well-being, with paw swelling reduced by 10.5% and tibiotarsal joint swelling reduced by 19.4% in comparison to untreated CIA rats at day 32 [73]. Post-transplantation arthritic symptoms were improved, including a 30% reduction in the arthritis index with radiological stabilisation revealed by X-ray radiographs based on cartilage and bone destruction, joint narrowing, and tissue swelling [73]. The histopathological investigation at 2 and 6 weeks after MSC-transplantation demonstrated improvement in synovial hyperplasia, reduced infiltration of inflammatory cells, and overall better joint condition in comparison to untreated CIA rats where thickening of synovial membrane, infiltration of lymphocytes, and polymorphonuclear cells and cartilage damage was reported [73]. These results were compatible with other studies [139]. Intravenous transplantation of umbilical cord blood (UCB)-MSCs into a CIA mouse model significantly reduced IL1β and IL6 protein expression by 19.4% and 42.4%, respectively, whilst increasing the expression of anti-inflammatory cytokine IL10 by 5.5-fold in paw tissues [139]. Treg populations were also shown to increase in a dose-dependent manner in CIA mice treated with UC-MSCs compared to the control group [139]. Transplantation of AT-MSCs into a CIA mouse model also demonstrated the suppression of T-cell autoimmune response, reduction in the clinical symptoms of arthritis, and decreased mean arthritic score, including erythema and paw swelling [134]. In this study, microcomputed tomography examined bone mineral density, trabecular bone volume fraction, trabecular number, thickness, separation, and connectivity density. Together the data revealed a significant reduction in bone loss and retention of trabecular bone architecture. The protection against bone loss was proposed to occur by MSC-mediated contact-dependent inhibition of the receptor activator of NFκB ligand (RANKL)-induced osteoclastogenesis in the presence of pro-inflammatory cytokines TNFα, IL17, and IL1β [134]. Slowing pro-inflammatory disease activity in arthritis models and activation of cartilage repair mechanisms provides evidence that MSCs may be used in cell-based therapies for the treatment of arthritis [73].

The therapeutic efficacy of MSCs has also been investigated in lupus nephritis in experimental mouse models. Meta-analysis of 28 studies evaluating the efficacy of MSCs demonstrated reduced levels of double stranded (ds)-DNA (odds ratios (OR), −29.58, 95% confidential intervals (CI) −29.58, −17.99, *p* < 0.0001), antinuclear antibody (OR, −70.93, 95% CI −104.55, −37.32, *p* < 0.0001), and proteinuria (OR, −4.26, 95% CI −5.15, −3.37, *p* < 0.0001) in the MSC treatment group against the control group [133]. The levels of IL2, IL12, and IL17 were significantly lower in the MSC treatment group compared with the control group (IL2: OR, −50.86, 95% CI −78.76, −22.96, *p* = 0.0004; IL12: OR, −328.24, 95% CI −652.20, −4.29, *p* = 0.05; IL-17: OR, −36.40, 95% CI −65.88, −6.93, *p* = 0.02). IFNγ was lower in the MSC group than in the control group (OR, −240.24, 95% CI −364.73, −115.75, *p* = 0.0002), and a comparable trend was shown with TGFβ, MCP1, and TNFα, although statistical significance was not achieved [133]. Lower renal sclerosis scores were recorded in MSC treatment groups compared with the control group (OR, −1.92, 95% CI −2.66, −1.18, *p* < 0.0001), suggesting that MSCs might be useful in the treatment of lupus nephritis [133].

MSCs have successfully promoted myelin repair in an experimental mouse model of autoimmune encephalomyelitis (EAE). Transplantation of BM-derived MSCs into myelin oligodendrocyte glycoprotein (MOG) 35–55-induced EAE demonstrated an 80% reduction in demyelination and a decrease in inflammatory cell infiltrates, including T-cells (50%), B-cells (51%), and macrophages (51%). This was coupled with a decline in disease progression measured by a 41% decreased cumulative score and a 60% lower maximal clinical score [74]. These results indicate that MSCs may be beneficial for the treatment of multiple sclerosis (MS) at the onset of disease when the immune response against myelin plays a major role in pathogenesis. MSCs derived from embryonic stem cells (ES-MSCs) have a greater neuroprotective potential than those derived from amniotic fluid (AF-MSC) and adult tissues and may therefore have a better therapeutic effect for the treatment of neurological diseases [135]. ES-MSCs showed a higher proliferative capacity in comparison to AF-MSCs, and higher anti-inflammatory potential due to increased NFκB-mediated release of anti-inflammatory cytokines [135]. Moreover, ES-MSCs injected into the brains of neonatal mice that had undergone hypoxic-ischemic insult showed significantly reduced microglial activation and transition of microglia to phagocytic phenotype. The loss of the cortex and pyriform cortex tissues was also reduced when compared to mice injected with AF-MSCs [135]. However, the risk of terataoma formation and ethical issues regarding the destruction of human embryos has nearly prohibited the clinical application of these ES cell derivatives [140].

## 7. Clinical Application of Mesenchymal Stem Cells in the Treatment of Autoimmune and Autoinflammatory Diseases

Following pre-clinical evaluation in experimental animal models, the therapeutic application of MSCs in the clinical setting has been considered for autoimmune and autoinflammatory diseases that currently have analgesic, i.e., symptom-alleviating, rather than curative treatments. Autoimmune and autoinflammatory diseases are mostly treated by immunosuppressants, but these are not always successful within a heterogeneous patient population. Continuous administration of medications can amplify side effects and long-term suppression of the immune system increases the risk of infections. Currently effective treatment options are limited and there is a need for new therapeutic approaches [141].

There is an historical context for the use of haematopoietic stem cell (HSC) transplantation that precedes MSC application. HSCs have been applied to poor prognosis and refractory treatment of severe autoimmune diseases since 1995. MSCs are considered as an attractive source for co-transplantation with HSCs because of their role in forming the microenvironment niche and their immunosuppressive properties that support allogeneic transplant viability. The first clinical application of BM-MSCs was performed in 1995, where the cells were used in the treatment of hematologic malignancy patients [142]. Since then, allogeneic or autologous MSCs have been used in the treatment of a multitude of severe diseases, including graft-versus-host disease (GVHD) [143]. Despite the extremely high level of mortality of GVHD, researchers recorded improved gut and liver measures including re-normalisation of bilirubin, liver biopsy histology, colonoscopy, and suppression of clinical manifestation which includes diarrhoea and abdominal pain. Objective improvement in clinical measures of GVHD has been demonstrated in 58% of gastrointestinal cases and 44% of liver cases when measured at day 28 post-MSC administration [143]. In addition, 76% of patients showed improvement in skin disease, with 44% of cases resolving completely [143].

Later, the efficacy of MSC treatment was proven in a phase II experimental trial for the treatment of leukaemia by co-delivery of MSCs with allogeneic HSCs. Results of the trial showed the ability of MSCs to modify innate and adaptive immune responses and provide an immunosuppressive effect that resulted in improved outcome measures for patients with steroid-resistant acute GVHD [144,145]. MSCs have now been used in the treatment of many autoimmune diseases, where standard therapeutic methods have proved ineffective (Table 4). BM has been considered to be the preferred tissue source for MSCs in therapeutic approaches, most likely because of the historical developmental pathway where BM-MSCs were first identified and characterised with relative abundance in BM tissue [3]. Experimental evidence suggests, however, that other tissue sources might be more therapeutically relevant for the treatment of autoimmune and autoinflammatory disorders. Thus, UC-MSCs and UCB-MSCs have many advantages compared to BM-MSCs since they are available in large quantities without invasive procedures and they have demonstrated good colony forming unit-fibroblast formation efficiency and greater immunomodulatory potential than BM-MSCs [146]. UC-MSCs were reported to have half the cell population doubling time and a higher number of population doublings than BM-MSCs [146]. They are considered to be more immunotolerant with a lower expression of HLA class I and an absence of HLA-DR even upon IFNγ stimulation, thus highlighting potential advantages over BM-MSCs [146]. However, there may also be donor-related MSC variability, which has been attributed to different factors that alter the metabolic environment in utero. The most relevant limitation is considered to be maternal obesity, which is accompanied by metaflammation. UC-MSCs from high BMI donors demonstrated slower population doubling but stronger immunosuppressive activity than MSCs derived from donors with lower BMI [147].

As well as exhibiting biological variation and heterogeneity of regenerative and immunomodulatory function, the source of tissue from which MSCs are derived is influential in the production of a cell-based therapeutic that can translate effectively to clinical application. For instance, invasive harvesting of tissues, including bone marrow, may not always be an appropriate option for patients compromised by inflammatory pain. Furthermore, MSCs derived from tissues affected by the pro-inflammatory environment of autoimmune and autoinflammatory disorders may not be of sufficient quality to effect repair [148,149,150].

**Table 4:** Clinical experience of MSCs transplantation in autoimmune diseases. A description of clinical studies of MSCs from different sources (including bone marrow (BM); umbilical cord (UC); adipose-tissue (AT)) and their application as a treatment of patients with autoimmune and autoinflammatory disorders using the following indicators of the efficacy: American College of Rheumatology 20% improvement criteria (ACR20);anti-cyclic citrullinated peptide (anti-CCP); anti-double-stranded DNA (anti-dsDNA); alkaline phosphatase (ALP); amyotrophic lateral sclerosis functional rating scale (ALSFRS); alanine transaminase (ALT); British Isles Lupus Assessment Group (BILAG); Derriford appearance scale (DAS24); 28-joint disease activity score (DAS28); Expanded Disability Status Scale (EDSS); gadolinium-enhancing lesions (GEL); gamma-glutamyl transferase (GGT); hospital anxiety and depression scale (HADS); health assessment questionnaire (HAQ); hepatitis B virus (HBV); hepatitis C virus (HCV); interleukin (IL); Model for End-Stage Liver Disease (MELD); magnetic resonance imaging (MRI); Systemic Lupus Erythematosus Disease Activity Index (SLEDAI); tumour necrosis factor-α (TNFα); visual analogue scales (VAS).

Disease	Patients (*N*)	MSC Type	Outcomes	Reference
Steroid-refractory acute graft-versus-host disease	55	Allogeneic BM-MSCs	More than half of the patients responded to the treatment measured by improvement in symptoms of acute GVHD. Patients had no side-effects.	[144]
Acute graft-versus-host disease resistant to multiple immunosuppressive agents in children	75	Allogeneic BM-MSCs	The rate of overall response (complete and partial response) was 66.7% for GVHD grade B, 76.2% for grade C, and 53.3% for grade D. Response for individual organs was 58.5% for the gastrointestinal system, 75.6% for the skin, and 44.4% for the liver. Overall response for patients treated for severe refractory GVHD was 61.3%, and this response was correlated with statistically significant improved survival at day +100 after MSC infusion.	[143]
Steroid-refractory acute graft-versus-host disease III/IV after hematopoietic stem cell transplantation	46	Allogeneic BM-MSCs	Clinical improvement in 50% (23/46) of patients: three patients (13%) had complete response, fourteen (61%) had partial response, and six (26%) had transient partial response. The estimated probability of survival at 2 year was 17.4%. Two patients (4.3%) presented acute transient side effects (nausea/vomiting and blurred vision) during cell infusion. No late or severe side effects.	[145]
Multiple sclerosis	20	Allogeneic UC-MSC	Improvement in EDSS scores (*p* < 0.03). Reduction in bladder, bowel, and sexual. dysfunction (*p* < 0.01), in non-dominant hand average scores (*p* < 0.01), in walk times (*p* < 0.02). MRI scans of the brain and the cervical spinal cord showed inactive lesions in 83.3% (15/18) patients after 1 year.	[151]
Multiple sclerosis	9 patients received MSCs (*N* = 5) or placebo (*N* = 4)	Autologous BM-MSCs	Patients treated with MSCs had lower mean cumulative numbers of GEL on MRI than in a placebo group after 6 months and reduced mean GEL after 12 months. Non-significant decrease in the frequency of Th1 (CD4^+^IFNγ^+^) cells in blood of MSCs treated patients. No serious adverse events.	[152]
Secondary progressive multiple sclerosis	10 patients had low-dose (1 × 10^6^ cells/kg) and 9 high-dose (4 × 10^6^ cells/kg)	Autologous AT-MSCs	One serious adverse event (one urinary infection—not related to study treatment). Measures for 12 months of treatment effect based on EDSS score and MRI were non-significant.	[153]
Amyotrophic lateral sclerosis	23	Autologous BM-MSCs	Reduction of ALSFRS decline at 3 months after application, in a few cases persisted for 6 months. 80% of the patients had stable forced vital capacity for a time period of 9 months and 60% of patients at 12 months after application. Weakness scales (WSs) remained stable in 75% of the patients at 3 months after application.	[154]
Amyotrophic lateral sclerosis	20	Autologous BM-MSCs	Statistically significant improvement in ALSFRS score. Improvement in forced vital capacity but insignificantly. Thirteen patients showed a 25% improvement in the slope of progression of ALSFRS-R (mean improvement of 47.4%, *p* < 0.0038). Three patients had an improvement of less than 25%. Three patients had a deterioration. No serious adverse events.	[155]
Rheumatoid arthritis	53	Allogeneic AT-MSCs	Persistent clinical benefit measured by ACR20, ACR50, low disease activity.	[156]
Rheumatoid arthritis	64	Allogeneic UC-MSC	The level of ESR, CRP, RF of 1 year and 3 years after treatment decreased. Anti-CCP of 3 years after treatment decreased. Health index (HAQ) and joint function index (DAS28) were lower 1 year and 3 years after treatment than before treatment. Liver and kidney function and immunoglobulin examination were normal.	[157]
Systemic lupus erythematosus with refractory cytopenia	35	BM-MSC	Significant improvement in leukopenia, anaemia, or thrombocytopenia. Reduction in proteinuria, antinuclear antibodies, and anti-dsDNA antibodies. Decline in disease activity according to SLEDAI score. Increase Treg, decrease Th17.	[158]
Systemic lupus erythematosus (severe and drug-refractory)	81	Allogeneic 22 BM-MSC, 59 UC-MSCs	84% survival rate (68/81 patients) after MSC. 27% of patients (22/81) in complete clinical remission. 7% (6/81) in partial clinical remission. 5-year overall rate of relapse of 24% (9/37). Serum albumin, peripheral leucocytes, and platelet number levels improved during fifth year of follow up. Decline in disease activity according to SLEDAI and remained significantly lower (*p* < 0.05) 5 years after MSC. Serum levels of complement three significantly increased (*p* < 0.05). 24-h proteinuria significantly decreased at 1-, 2-, 3-, 4-, and 5-year follow-up (all *p* < 0.05).	[159]
Lupus nephritis	18 patients received MSCs (*N* = 12) or placebo (*N* = 6)	Allogeneic UC-MSCs	Remission occurred in 75% of patients (9/12) in the UC-MSC group, in comparison to 83% of patients (5/6) in the placebo group. Mean time to remission was 9 weeks for the UC-MSC group and 16 weeks for the placebo group. 3.2-fold reduction in proteinuria at 6 months in the UC-MSC group compared with 1.4-fold reduction in proteinuria in the placebo group. Improvement in the SLEDAI and BILAG scores, anti-dsDNA antibody, and ANA and serum C3 and C4 concentrations with no difference between groups. Serum creatinine remained stable in both groups.	[160]
Systemic sclerosis	14	Allogeneic UC-MSCs	Reduction of modified Rodnan skin score. Improvement in lung function and computed tomography after 12 months of combined therapy. Decrease in the anti-Scl70 autoantibody, TGFβ, and vascular endothelial growth factor.	[161]
Systemic sclerosis	62	Autologous AT-MSCs	Significant 22% improvement in mouth function. Improvement in the psychological status: 15% decrease in VAS and 22% decrease in DAS24 scores. Decrease in the level of psychological distress related to physical appearance: 27% improvement in HADS-A score that measures levels of anxiety, 24% decrease in HADS-D score that measures levels of depression. Reduction in SSc fibroblast viability and proliferation was significant after 14 days of co-culture with AT-MSCs. Decrease in TGFβ1 and connective tissue growth factor in co-culturing SSc fibroblasts with AT-MSCs. Decrease in Matrix metalloproteinase-8, Platelet derived growth factor-β, and Integrin Subunit Beta-6 in SSc co-culture with AT-MSCs compared to monoculture after 14 days.	[162]
Liver cirrhosis caused by autoimmune diseases (mixed connective tissue disease, primary biliary cirrhosis, primary Sjögren’s syndrome, rheumatoid arthritis, systemic lupus erythematosus, systemic sclerosis)	26	Allogeneic (23 patients received UC-MSCs, 2 received cord blood MSCs and 1—BM-MSCs)	ALT, ALP, GGT, and total bilirubin decreased. Average serum albumin levels improved. Improvement in Model for End-Stage Liver Disease (MELD) scores.	[163]
Idiopathic pulmonary fibrosis	8	Allogeneic placenta-derived MSCs	Slight improvement in all spirometry tests. Fibrosis scores were unchanged—no evidence of worsening fibrosis.	[164]
Idiopathic pulmonary fibrosis	9	Allogeneic BM- MSCs	No serious adverse events. Two nontreatment-related deaths occurred because of progression of IPF (disease worsening and/or acute exacerbation). Recorded 3.0% mean decline in % predicted forced vital capacity and 5.4% mean decline in % predicted diffusing capacity of the lungs for carbon monoxide by 60 weeks after MSC transplantation.	[165]
COVID-19	7 (1 critically severe type, 4 severe types and 2 common types)	Autologous BM-MSCs	The pulmonary function and symptoms of all patients were significantly improved at 2 days after transplantation. Two common and one severe patient were recovered. Peripheral lymphocytes level increased. CRP decreased. Overactivated cytokine-secreting immune cells CXCR3^+^CD4^+^ T-cells, CXCR3^+^CD8^+^ T-cells, and CXCR3^+^ NK cells disappeared in 3–6 days. CD14^+^CD11c^+^CD11b^mid^ regulatory DC cell population increased. The level of TNF-α decreased, while the level of IL10 increased.	[166]

Clinical trials have investigated the safety and efficacy of MSCs in the treatment of inflammatory kidney diseases, including nephritis associated with lupus and diabetes, autosomal dominant polycystic kidney disease and atherosclerotic renovascular disease [159,160]. Intravenous transplantation of allogeneic BM- and UC-MSC in severe and drug-refractory SLE patients demonstrated statistically significant improvement in proteinuria, serum albumin, complement C3, peripheral leucocytes, and platelet numbers at 24-h post-infusion. There was also a significant decline in disease activity measured against the systemic lupus erythematosus disease activity index (SLEDAI) at the fifth year of follow-up [159]. The 5-year overall survival rate of patients with severe drug-refractory SLE after MSC transplantation was 84% (68/81 patients), with 27% of patients (22/81) achieving complete clinical remission and 7% of patients (6/81) achieving partial clinical remission [159].

In another study, SLE patients with refractory cytopenia treated with BM-transplantation demonstrated a significant improvement in blood cell count (leukocytes, erythrocytes, thrombocytes), and this was accompanied by a 43.65% reduction in SLEDAI at 3-months and 72.44% at 24-months follow-up [158]. Immune cell populations were also reported to be moderated with a 53.7% increase in Treg cells and a 54% reduction in Th17 cells at 1-month post-BM-MSC transplantation [158]. However, data obtained from another randomised double-blind placebo-controlled trial of allogeneic UC-MSCs transplantation for the treatment of lupus nephritis showed no additional therapeutic benefit of MSCs under standard immunosuppression, including intravenous methylprednisolone and cyclophosphamide or oral prednisolone and mycophenolate mofetil therapy [160]. Following transplantation of UC-MSC at a dose of 2 × 10^8^ cells, 75% (9/12 patients) achieved remission with a reduction of haematuria and proteinuria. This was achieved in comparison to 83% of patients (5/6) in the placebo group. Overall, the study revealed an improvement in the SLEDAI and in the British Isles Lupus Assessment Group (BILAG) scores in both groups [160]. Examination of anti-dsDNA antibody, ANA, and serum C3 and C4 concentrations did not show any difference between groups [160].

Analysis of eight pilot trials in which MSCs were co-delivered with renal transplantation showed prolonged graft survival and reduction in dose of immunosuppressive drugs, including tacrolimus, mycophenolate mofetil, or cyclosporin A, and this was predicted to be a result of the immunosuppressive, anti-oxidative, and reparative-regenerative properties of MSCs [167].

The efficacy and safety of MSCs of different cell origins (UC-MSCs, BM-MSCs, stromal vascular fraction-MSCs) in the treatment of SSc has been demonstrated in nine clinical studies, including 133 adult patients [168]. Systematic review and meta-analyses of these research data showed reduction of the modified Rodnan skin score (mean difference (MD) 5.23, 95% confidential intervals (CI) 4.18–6.29, *p* < 0.00001) and significant decrease in the number of digital ulcers after 6 months of treatment with MSCs (odds ratios (OR) 21.10, 95% CI = 3.63–122.56, *p* = 0.0007), as well as visual analogue scale of hand pain in SSc patients (MD = 7.09, 95% CI 0.53–13.65, *p* = 0.03). However, Raynaud’s phenomenon score and Cochin hand function scale score were not changed significantly at 6 months of MSCs therapy (MD = 1.8, 95% CI − 3.38 to 6.99, *p* = 0.50). Zhang et al. (2017) demonstrated that combined therapy, including plasmapheresis, pulse cyclophosphamide, and allogeneic UC-MSCT resulted in 31% improvement in Rodnan skin score, improvement in carbon monoxide diffusing capacity, and forced vital capacity of SSc patients (*p* < 0.05) at 12 months of follow-up [161]. Serological changes such as a 51.32% reduction in Anti-Scl70 autoantibody and a 47.09% decrease in VEGF were also found after a 12-month follow up period [161].

Assessment of 62 patients with SSc treated with autologous AT-MSCs revealed a 22% improvement in mouth function measured by the Mouth Handicap Scale as well as enhancement in psychological status determined by VAS score [162]. The study also demonstrated a significant reduction in the viability and proliferation of dermal fibroblasts derived from SSc patients following co-culture with AT-MSCs for 14 days (*p* < 0.0001). This effect was associated with a decrease in TGFβ1 and connective tissue growth factor (CTGF) production, and reduced expression of fibrosis-associated genes, including matrix metalloproteinase-8 (MMP-8) and integrin Subunit Beta-6 (ITGβ6) [162].

In the area of arthritis, the first studies to investigate MSCs were in patients with RA who had not responded to conventional pharmaceutical therapy. Studies investigating the role of allogeneic BM-MSCs and UC-MSCs by infusion into patients with RA have demonstrated a moderate response according to EULAR criteria [156,157]. Sixty-four RA patients who underwent UC-MSCs therapy combined with DMARDs demonstrated reduction in HAQ and DAS28 scores, as well as reduction in C-reactive protein (CRP), ESR, and anti-cyclic citrullinated peptide (anti-CCP) at 1-year and 3-year follow-up [157]. Clinical efficacy was maintained for 3 years post-MSCs transplantation without any serious side effects reported during or after UC-MSCs infusion [157].

The treatment of paediatric rheumatic diseases with MSCs has also been investigated in patients who previously had no response to all currently available treatment options, including biologics. AT-MSCs transfused into a child with SLE refractory to standard therapy resulted in a decrease in global assessment PGA from 8/10 to 1/10, ANA declined from 1:640 to 1:80, and the patient become clinically stable for 2 years [169]. Allogeneic UC-MSCs transplanted into a patient with juvenile idiopathic arthritis (JIA) had improved PGA from 6/10 to 1/10 and Juvenile Arthritis Disease Activity Scores (JADAS) from 11 to 6 [169]. A single-centre open label intervention study in six patients with JIA resistant to biological therapy reported a 25% decrease in VAS well-being (*p* = 0.043) and a 55.1% decline in the JADAS-71 (*p* = 0.043) at 8 weeks post-allogeneic BM-MSC transfusion compared to the start of the study [170]. One year after MSC transplantation, the patients had significantly lower active joint count, VAS well-being, VAS pain, physician global assessments, cJADAS-10, JADAS-71, and Quality of Life (from JAMAR) scores than at the start of the study (*p* < 0.046) [170]. However, one patient with systemic onset JIA (sJIA) had a relapse of macrophage activation syndrome (MAS) at 7 weeks post-MSC infusion and 9 weeks after tocilizumab discontinuation [170]. Thus, MSC may be a powerful tool in the therapy of childhood rheumatic disease, since they were well tolerated with no serious adverse events such as ectopic growth, emboli, or malignancy in the examined children [169], but ceasing biologic treatment may increase the risk of a MAS flare [170]. This highlights the need for well monitored controlled clinical trials with MSCs in paediatric rheumatic disease.

Intravenous infusion of UC-MSC for the treatment of multiple sclerosis (MS) showed an 11.7% reduction in disease activity measured by the Kurtzke Expanded Disability Status Scale (EDSS) test, and a 2% decline in the Scripps Neurological Rating Scale with significant improvement in bladder, bowel, and sexual function [81]. In addition, an increase in non-dominant hand average scores and in walk times (*p* < 0.02) were registered after 1 year compared to baseline [151]. MRI scans of the brain and the cervical spinal cord demonstrated no disease progression or no new or active lesions in 83.3% patients at 1-year post-treatment [151]. In another study, patients with MS who were unresponsive to conventional therapy demonstrated a four-fold reduction in the mean cumulative number of gadolinium-enhancing lesions (GEL) on an MRI scan at 6 months post-BM-MSC transplantation, but there was no significant improvement in the EDSS [152]. Clinical measurements were correlated with a modest reduction in Th1 and Th17 lymphocytes and an increase in Breg populations in the peripheral blood of MSC-treated patients in comparison to the control group [152]. In contrast, Fernández et al. (2018) reported that intravenous delivery of AT-MSCs showed no statistical improvement in clinical outcome measures, including number of relapses, EDSS score, and MRI non-normalised cerebral volume or number of active lesions in Gd-enhanced T1 scans [153].

In a phase II clinical trial for amyotrophic lateral sclerosis (ALS), repeated dosing of autologous BM-MSCs via intrathecal transplantation showed a statistically significant improvement in Amyotrophic Lateral Sclerosis Functional Rating Scale Revised (ALSFRS-R) score [155]. The treatment protocol for this research was intended to include MSC injections every 3 months during 2 years, however, due to low number of cells or the unwillingness of the patients to undergo repeated lumbar punctures the treatment intervals were extended individually and patients received MSCs between 1 and 4 times. Of the MSC-transplanted ALS patients, the majority (65%) demonstrated a greater than 25% slower rate of progression along the ALSFRS-R after MSC transplantation compared with the pre-treatment period (mean improvement of 47.4%, *p* < 0.0038) [155]. Another prospective, non-randomised, open-label clinical trial showed a slowing of ALS progression at 3 months (*p* < 0.001), as well as at 6, 9, and 12 months (*p* < 0.01), with reduction in ALSFRS decline following BM-MSCs transplantation into the cerebrospinal fluid of 23 patients [154]. Forced vital capacity (FVC) and values of weakness scales remained stable for a period of 9 months [154].

Between 2020–2023, MSCs have been used as a potential therapy for treating patients with severe SARS-CoV-2-associated inflammation [171]. The first report from a pilot trial was obtained from China, where seven patients with COVID-19 pneumonia received MSC transplantation with assessment up to 14 days post-treatment [166]. At 2–4 days post-transplantation, clinical symptoms, including high fever and shortness of breath were reduced and blood oxygen saturation was increased to ≥95% at rest [166]. Based on satisfactory clinical results the authors concluded that MSCs could improve the outcomes of COVID-19 without any transfusion side effects. Up-to-date meta-analysis of MSC treatment of COVID-19 revealed that intravenous infusion of UC-MSC significantly decreased the risk of mortality in comparison to the control group (*p* = 0.03) [171]. No statistical significance was observed in the incidence of adverse events (*p* = 0.44). The ability of MSC in reducing inflammatory response was not determined because the levels of CRP or IL6 changed insignificantly (*p* = 0.06 and *p* = 0.09, respectively) [171].

## 8. Risks and Challenges of Stem Cell Transplantation

MSCs have been widely investigated in the treatment of several very severe refractory inflammatory diseases and has included thousands of participants with GVHD, MS, ALS, RA, and SLE. Treatment-related adverse events associated with MSC administration have been evaluated by systematic reviews. One of the biggest meta-analysis projects to review MSC safety included 62 randomised clinical trials involving 3546 participants and highlighted an association with transient fever at 48 h post-MSC administration (odds ratios (OR), 3.65, 95% confidential intervals (CI) 2.05–6.49, *p* < 0.01), and adverse events at the administration site including injection site bleeding, swelling, itchiness, pain, or local infection (OR, 1.98, 95% CI 1.01–3.87, *p* = 0.05) [172]. Minor adverse events associated with MSC transplantation were sleeplessness (OR, 5.90, 95% CI 1.04–33.47, *p* = 0.05), fatigue (OR, 2.99, 95% CI 1.06–8.44, *p* = 0.04), and constipation (OR, 2.45, 95% CI 1.01–5.97, *p* = 0.05) [172].

Other side effects have been reported and include the presence of acute transient side effects such as nausea/vomiting and blurred vision during MSC infusion in 2 of 46 patients with steroid refractory GVHD (4.3%) [145]. Thromboembolism induced by stem cell transplantation was described in two patients with renal transplantation and chronic kidney disease although the total cohort size was not reported [173]. MSC infusion caused venous obstruction and swollen extremities, but in these cases thrombosis was successfully treated with urokinase and warfarin thrombolytic therapy [173].

The oncological risks of MSC transplantation have been widely discussed because of their high proliferative capability and theoretical potential for malignant transformation. MSCs are attracted to injured tissues and wounds, but also may be recruited to tumours in response to the over production of growth factors (PDGF, HGF), cytokines (IL1β, IL8, TGFβ, TNFα), angiogenic factors (such as VEGF), and some chemokines (CCL5, CCL2, CXCL12, and CCL22) [174]. MSCs recruited to the tumour microenvironment in response to hypoxia or pro-inflammatory cytokines, including IL1β, TNFα, and IFNγ, form tumour-associated MSCs, which have been shown to further transdifferentiate to cancer-associated fibroblasts (CAFs). CAFs secrete pro-angiogenic and immunosuppressive factors, including PDGF, FGF, VEGF, and IL6 and IL8, which go on to contribute to cancer cell survival, ‘stemness’, angiogenesis, and immunosuppression, and the promotion of tumorigenic growth and metastasis [174,175]. CAFs are formed in response to TGFβ and FGF production in the tumour microenvironment, acquiring an expression profile that includes αSMA, fibroblast activation protein, thrombospondin-1, tenascin-C, desmin-1, and VEGF-AA, and as a terminally committed cell type are unable to return to their naïve phenotype or undergo apoptosis. CAFs contribute to the recruitment of monocytes and M2 macrophage polarisation to M2 [174]. However, the majority of studies investigating the conversion of MSCs into CAF subtypes were carried out in vitro and were therefore dependent on different culture conditions, including continuous inflammatory stimulus by TNFα and IL1β [129,176]. Further studies including the identification of potential CAF markers may advance the understanding of the mechanisms, and hence the actual risk of MSC modification into CAFs within a native in vivo environment and when delivered as a cell-based therapeutic.

The key question is whether the generation of tumorigenic cells is a result of ex vivo MSC expansion in culture. Senescent MSCs that have exited a cell cycle obtain a senescence-associated secretory phenotype (SASP) characterised by the secretion of a cocktail of pro-inflammatory cytokines (IL6, IFNγ, TNFα), chemokines (IL8, MCP1), growth factors (FGFb, HGF, GM-CSF), proteases (MMPs, TIMP-2), soluble adhesion molecules and cell surface receptors (ICAM, VCAM, EGFR), extracellular matrix (ECM) components (fibronectin, laminin), some non-protein small molecules (NO, PGE2), growth-related oncogene (GRO), and macrophage-derived chemokine (MDC). The SASP is also associated with systemic inflammation and is responsible for a paracrine-mediated ‘bystander effect’ in which surrounding cells are induced to senescence, amplifying the pathophysiologic response to tissue dysfunction [177]. The composition of SASP, which is released by damaged or senescent fibroblasts, is known to support tumour growth [178]. Other research demonstrated that SASP may block the proliferation, as well as induce the growth arrest and apoptosis of cancer cells [179]. Finally, Alessio et al. (2019) showed that SASP derived from MSCs that had undergone senescence by treatment with hydrogen peroxide or with low X-rays could induce senescence in immortalised prostate cells and therefore may be considered as an effective agent against pre-tumorigenesis [180]. Prolonged in vitro expansion affects the immunomodulatory efficacy of MSCs because of the progression of replicative senescence. MSC senescence is mediated by p53/p21, p16/RB, p38MAP kinase, mitogen activated protein, and signal transducer and activator of transcription-3 (STAT3) signalling pathways, leading to a permanent cell cycle arrest, altered autophagy homeostasis, and irreversible DNA damage that manifests in dysfunctional immunomodulation of immune cell responses [181]. Phenotypic changes associated with replicative senescence include morphological alterations (loss of fibroblastic morphology and enlarged cell volume), reduction of proliferation rate, impaired differentiation and homing capacity, and mitochondrial dysfunction. Changes in the secretory phenotype from anti-inflammatory to a pro-inflammatory secretome are also reported [181]. Another mechanism includes in vitro progressive telomere shortening and induction of genomic instability, which occurs during multiple cell culture passages [181]. Epigenetic modifications reduce ‘stemness’, evidenced by the reduced capacity for self-renewal and differentiation and are concomitant with the downregulated expression of cell surface markers associated with the MSC phenotype, including stromal cell surface marker-1 (STRO1), CD106, and CD146 (MCAM) [181]. As well as long-term in vitro culture, the age of the donor may also be causal to genetic instability and chromosomal aberrations, elevating the risk of cell transformation and tumour formation [182]. To reduce these risks, genetic characterisation of MSC populations by conventional karyotyping and molecular array-comparative genomic hybridisation has been proposed to identify potential chromosomal abnormalities in cultured MSCs prior to clinical application [182].

## 9. Comparison of Allogeneic and Autologous Sources of Mesenchymal Stem Cells

Debate over the benefit of allogeneic or autologous MSC therapy has been widely discussed [115,183], with the proposition that allogeneic MSCs are more advantageous than those harvested from autologous sources [183]. A higher quality of MSCs may be acquired from allogeneic sources because of the ability to control patients’ age and health status, cell potency, and absence of genetic and epigenetic abnormalities (Table 5). The disadvantages of allogeneic MSCs have been shown with reports that these cells are not absolutely immune-privileged and despite low expression of MHC class I and II, can still be recognised by immune response and rejected after about 20 days in vivo [81].

The process of cryopreservation has important implications on the efficiency of clinical translation of MSC-based therapies. Application of allogeneic therapies will enable the production of ‘off-the shelf’ products, minimising the number of surgical interventions undertaken by the patient and maximising the number of therapeutic products that can be manufactured per tissue donor. For autologous applications, MSCs can be harvested from healthy tissues and cryopreserved when required at a later date. To achieve this, more information is required regarding the impact of cryopreservation on the biological status of the cells, and by extension how both safety and efficacy is affected for both allogeneic and autologous applications [184]. It has been reported that cryopreservation of allogeneic MSCs can alter the survival of MSCs when recovered from cryopreservation in comparison to fresh MSCs in a model of normothermic machine perfusion to support transplant kidneys [185]. In addition, cryopreserved MSCs were characterised by elevated levels of ROS and reduced mitochondrial activity [185]. This points to an enhanced level of oxidative stress and indicates impaired metabolism of MSCs following cryopreservation. Autologous MSC transplantation is considered to be ‘safer’ than the use of allogeneic cells, but harvesting of autologous MSCs requires time for in vitro cell expansion and additional previous stimulation or surgery [115]. The quality and quantity of these cells may be lower than those derived from an allogeneic source, due to the presence of disease or the age of the patient (Table 5).

The age of the donor is also an important parameter that restricts the benefit of autologous MSCs transplantation. MSCs taken from older patients are known to have higher levels of replicative senescence, evidenced by significantly fewer CFU-Fs formed on derivation, reduced proliferation rate, reduced immunomodulatory properties, and an increased pro-inflammatory phenotype compared to those derived from younger donors [186,187,188]. These studies have shown that MSCs derived from elderly donors have lower superoxide dismutase activity and increased production of nitric oxide and ROS, leading to oxidative damage and senescence [188]. Hallmarks of senescence, including SA-β-gal expression, were higher in AT-MSCs obtained from patients in the elderly donor group (>50 years) compared to patients in the young donor group; 12.2 ± 1.1% vs. 5.2 ± 1.9% SA-β-gal positive cells; *p* < 0.05) [187], and the expression of senescence-associated p16 and p21 genes was also significantly higher in MSCs from elderly donors (>50 years) when compared to younger donors (<40 years) (*p* < 0.05) [187].

Similarly, autologous MSCs derived from patients with autoimmune or autoinflammatory diseases may have a compromised genetic background that predisposes their stem cell compartment to immune disorders. An example of this is evident in juvenile idiopathic arthritis (JIA) where both HLA and non-HLA-related genes are heavily influential in pre-disposing disease susceptibility [189]. For these conditions, the use of allogeneic MSCs has been considered as a more preferable option for safe and effective treatment.

Considering the low expression of MHC class II antigens and the lack of the immune co-stimulatory receptors, allogeneic MSCs do not provoke a strong immune response and probably can be used for the treatment of diseases without complications. Many systemic intravascular delivery and intra-articular injections of autologous or allogenic MSCs have been performed over the last decade, without any serious complications, such as malformation or sepsis [119,157]. However, it is important to consider that the immunogenicity of MSCs may change under the influence of pro-inflammatory environments into which they are delivered, with pro-inflammatory mediators at sites of inflammation stimulating the upregulation of immune molecules, including MHC Class II [115]. To understand with certainty if MSC transplantation is beneficial for treatment of all types of autoimmune and autoinflammatory diseases, where there are a spectra of pro-inflammatory mediator compositions, future research needs to focus on long-term clinical trials that investigate changes in MSC phenotype and function following transplantation.

To summarise the results obtained from the preliminary analysis of studies of MSC transplantation, the potential risk may be defined from the allergic reactions in response to bovine proteins (safety of medium), ectopic tissue formation or malignant transformation, infection, aggregation of the cells, and embolisation. Nevertheless, in clinical trials in adult and paediatric populations, all complications of MSC therapy, except fever and adverse events at the administration site, did not correlate with cell transplantation [119]. A major step toward adoption of MSC therapies came in 2018 with the first allogeneic MSC product approved for use in the European Union [190]. It remains that some questions are still open to be addressed. One of them is the functional heterogeneity of MSCs and their plasticity of response when stimulated by complex combinations of bioactive factors, all of which can have an impact on the therapeutic outcome of the MSC product. The safety and efficacy of MSCs in clinical application depends not only on the biological properties of the cells but also on the microenvironmental factors within the site into which the cells are being transplanted, for instance the inflammatory status of the tissue. There is therefore a need to develop strategies beyond standardisation of the phenotype and functional properties of MSCs, such as optimisation of bioprocessing and delivery protocols. Further work is required to explore the complexity of the tissue environments into which the cells are to be transplanted, so as to be able to predict the functional response of the cells when they are transplanted [115]. Ultimately, this work should be progressed to open-label multi-centre clinical trials that measure and evaluate the long-term follow-up of MSC transplantation in order to verify their efficacy and safety in the treatment of autoimmune diseases.

## 10. Conclusions

Taken together, the immunomodulatory and regenerative properties of MSCs, driven by direct cell contact or production of exosome secretions, place these cells as important candidates for potential clinical application in the treatment of autoimmune and autoinflammatory diseases. However, contemporary studies have shown that MSCs obtained from patients with these pathologies have impaired biology that restricts proliferative, differentiation, and immunomodulatory properties. Further research is required to form a comprehensive understanding of the contribution that MSCs make to the pathogenesis of autoimmune and autoinflammatory diseases and their application as therapeutics for moderating immune responses in clinical cases where standard therapeutic methods have proved ineffective.

## Figures and Tables

**Table 1:** Bioactive molecules that have a role in immune homeostasis. The table describes key growth factors and cytokines that have a role in the homeostasis of immune cell responses and the pathogenesis of autoimmune and autoinflammatory disorders.

Growth Factor	Role in Immune Regulation Inflammation and Disease	Reference
Monocyte chemotactic protein-1 (MCP1)	Increased expression by stromal and immune cells triggered via NF-κB-mediated response to pathogen-associated and molecular-associated molecular patterns released by damaged cells. Promotes upregulation of chemokine receptor expression and infiltration of immune cells to tissues. Acts as a key cytokine in age-related senescence-associated secretory phenotype (SASP), and contributes to ‘inflammaging’ by propagation of pro-senescent signals through the tissue and promotion of chronic inflammation associated with chronic disease.	[14,15,16,17,18]
Macrophage migration inhibitory factor (MIF)	Fundamental to the pro-inflammatory response being released by immune cells in response to pathogen-associated molecular patterns. Propagates inflammatory response by autocrine and paracrine stimulation of pro-inflammatory cytokine release. Elevated expression in sepsis correlating with cortisol and IL6 expression and prognosis of disease progression. Upregulated in acute respiratory distress syndrome where it is directly linked to promotion of the inflammatory response and production of pro-inflammatory cytokines.	[19,20,21,22,23,24,25,26,27,28,29]
Basic fibroblast growth factor-2 (FGF2)	Regulator of cellular activity during tissue repair and regeneration, including mediation of inflammatory response during the acute phase of injury. Promotes upregulation of pro-inflammatory cytokines in immune cells and tissue-resident somatic cells. Increased expression associated with inflammation results in tissue fibrosis, contributing to inflammaging and impairment of tissue function that manifests as age-related chronic diseases and disorders.	[30,31]
Vascular endothelial growth factor (VEGF)	Increased expression during inflammatory response to promote angiogenesis and support the infiltration of immune cells. Contributes to the regulation of adhesion molecule expression to control the infiltration of immune cells across capillaries. Increased levels work with HIF1α, angiopoietins, TNFα, and IL8 to promote angiogenesis. Angiogenesis and microvesicle remodelling are a hallmark of inflammatory associated diseases, including psoriasis, RA, inflammatory bowel disease, and diabetic retinopathy.	[32,33,34,35,36]
Hepatocyte growth factor (HGF)	Expressed in immune cell organs, including bone marrow, thymus, tonsils, and spleen with a key role in supporting haematopoiesis and immune cell development. Elevated expression during regeneration of tissues in response to a pro-inflammatory environment and particularly cytokines IL1α, IL1β, TNF, and interferon (IFN)-γ. Dysregulation of HGF activity is implicated in inflammatory disorders through overstimulation of T-cells and production of pro-inflammatory cytokines, maturation of monocytes to macrophages, and migration of dendritic cells.	[37,38,39,40,41,42,43,44,45,46]
Insulin-like growth factor-1 (IGF1)	Anti-inflammatory cytokine widely expressed by immune cells. Regulates macrophage polarisation from pro-inflammatory M1 phenotype to anti-inflammatory M2 phenotype. Shift from M1 to M2 macrophage polarisation is proposed as being protective against autoimmune and autoinflammatory disorders but its overexpression may be explicit in the progression of fibrosis.	[47,48,49,50]
Platelet-derived growth factor (PDGF)	Expressed by monocytes and platelets with increased expression in response to injury where it moderates immune cell activity, including inhibiting dendritic cell cytotoxic activity, and modulation of macrophage and lymphocyte activity. Reduced PDGF levels during the early inflammatory phase of arthrosclerosis result in increased monocyte and pro-inflammatory T-cell presence within developing lesions.	[51,52,53,54,55]
Transforming growth factor-β1 (TGFβ1)	Key growth factor in the maintenance of immune cell homeostasis. Stimulates pathogenic Th17 cell differentiation in combination with IL6, IL1, and IL23 and is a potent mediator of autoimmune disorders.	[56,57]
Stromal cell-derived factor-1/C-X-C motif chemokine-12 (SDF-1α/CXCL12/)	Regulates immune cell trafficking with dysfunction causing pathological recruitment and retention of immune cells to tissues and progression of autoimmune and autoinflammatory disorders. Contributes to the chronic inflammation of inflamed joints in RA disease by promotion of activated immune cell homing and retention within the joint. Directly promotes joint tissue erosion by promoting migration and maturation of osteoclasts, inducing chondrocyte necrosis and promotion of neovascularisation. Elevated expression in inflammatory psoriasis with contribution to promotion of angiogenesis in skin lesions. Elevated expression in cerebral spinal fluid, astrocytes, and monocytes/macrophages of active lesions in patients with multiple sclerosis.	[58,59,60,61,62,63,64,65,66]

**Table 2:** Immunomodulatory properties of MSCs. The table summarises the diverse mechanisms by which MSCs perform their immunomodulatory functions via cell–cell contact or paracrine effects. Dendritic cells (DCs); interferon gamma (IFNγ);indoleamine 2,3-dioxygenase (IDO); interleukin (IL); hepatocyte growth factor (HGF); leukaemia inhibitory factor (LIF); natural killer cells (NK); nitric oxide (NO); prostaglandin-E2 (PGE2); soluble human leukocyte antigen G (sHLA-G); transforming growth factor-beta (TGFβ); tumour necrosis factor-alpha (TNFα); regulatory T-cells (Treg); vascular endothelial growth factor (VEGF).

Property of MSC	Mechanism
Suppression of T-cell activity	Inhibition of antigen-specific proliferation (both for naive and memory T-cells). IFNγ and IL4 production. Arrest of T-cells in the G0/G1 cell cycle phase.
Inhibition of B-cells	Block of activated B-cell proliferation. Decrease in antibody production. Suppression of B-cell chemotaxis by reducing surface expression of the chemokine receptors on B-cells.
Activation of regulatory T-cells	Increase production of sHLA-G, inducing the differentiation of Treg-cells. Induction of Tregs is caused by cell-to-cell contact with MSCs and by the secretion of PGE2 and TGFβ1.
Inhibition of NK cells	Production of TGFβ, sHLA-G, and PGE2. Cell–cell contact inhibits NK cell cytotoxicity.
Induction of macrophages with anti-inflammatory immunophenotype	PGE2 induction of macrophages to produce IL10. Phagocytosis of dead MSCs by macrophages leads to appearance of alternatively activated macrophages characterised by increased production of IL10, TGFβ3, and IL6, and decreased TNFα and IL12 secretion. MSC-educated macrophages have increased expression of alternatively activated macrophages markers CD206 and CD163 and the inhibitory molecules PD-L1 and PD-L2.
Regulating lymphopoiesis	BM-MSC regulate the development of T- and B-lymphocytes through the action of growth factors, cytokines, and adhesion molecules.
Interaction with DC	MSCs negatively regulate DC differentiation from CD14^+^ monocytes and CD34^+^ progenitor cells by altering the expression of the DC surface antigens and IL12 production.
Paracrine effects of MSCs	Secretion of growth factors, anti-inflammatory cytokines, chemokines, (IL10, IL6, TGFβ, VEGF, sHLA-G, HGF, IDO, NO, PGE2, and LIF). Suppression of pro-inflammatory cytokine (IFNγ, IL1β, TNFα) production. Extracellular vesicles contain bioactive molecules, including mRNA and miRNA and mitochondria.

**Table 3:** Morphological and physiological impairment in MSCs in the pathogenesis of autoimmune and autoinflammatory diseases. The table describes autoimmune and autoinflammatory disease with the methodological summary used to characterise associated MSC dysfunction. Amyotrophic lateral sclerosis (ALS); ankylosing spondylitis (AS); bone marrow (BM); chemokine (C-C motif) ligand-2 (CCL2); carboxyfluorescein diacetate succinimidyl ester CFSE); colony-forming unit–fibroblast (CFU-F); chronic obstructive pulmonary disease (COPD); cyclooxygenase-2 (COX2); endothelial-like MSC (EL-MSCs); indoleamin-2,3-dioxygenase (IDO); idiopathic pulmonary fibrosis (IPF); hepatocyte growth factor (HGF); mesenchymal stem cell (MSC); peripheral blood mononuclear cells (PBMCs); programmed death ligand-1 (PDL1); rheumatoid arthritis (RA); transforming growth factor-β (TGFβ); TNF-stimulated gene-6 (TSG6); synovial fluid (SF); systemic lupus erythematosus (SLE); systemic sclerosis (SSc); T-helpers (Th); T-regulatory cells (Treg); visual analogue score (VAS); vascular endothelial growth factor (VEGF); vascular endothelial growth factor receptors (VEGFRs).

Disease	Study Methodology	MSC Characteristics	References
Rheumatoid arthritis (subtype was not defined despite clinical importance and is a limitation of the study)	Synovial inflammation measured using the arthroscopic visual analogue score (VAS) and by immunohistochemistry with anti-CD3 and CD68 staining for macrophages. Expression of SOX9, p65, Galectin-3, and SUMO measured by qPCR. Synovial MSCs were analysed by population doublings, clonogenic activity, and multipotency. ELISA for IL1 β, TNFα, IL6.	The arthroscopic VAS in RA correlated significantly with synovial macrophage infiltration. RA activity negatively influences synovial MSC by decreasing their chondrogenic and clonogenic capability. CD44 in RA MSCs correlated negatively with inflammation and positively with chondrogenesis. Cytokine production and Sox9 expression was similar in RA MSCs and OA MSCs.	[126]
Rheumatoid arthritis (subtype was not defined despite clinical importance and is a limitation of the study)	Co-culture of BM-MSC, CD4^+^ cells, or PBMCs labelled with CFSE and measurement of Th-cells, Treg, and Th17-cells by flow cytometry. Proliferation and apoptosis assays. Migration assays. Human G-Series Cytokine Antibody Array. ELISA measured of IL17A. TGFβ1, IDO, PGE2, IL6, and CCL2 measured by qPCR.	RA MSCs showed equivalent immunophenotype, differentiation potential, cellular apoptosis, and cytokine profiles compared to controls which were OA patients who underwent knee arthroplasty. BM MSCs from RA patients did not downregulate Th17-cells proliferation. RA derived-MSCs showed impaired proliferative potential and migration capacity.	[122]
Ankylosing spondylitis	Multiple differentiation and cell viability assays. Immunomodulatory property of MSCs were analysed by two-way mixed PBMCs reactions or after stimulation with phytohemagglutinin. CCR4^+^CCR6^+^ Th/Treg cells and surface markers of BM-MSCs were analysed using flow cytometry.	AS MSCs demonstrated normal proliferation, cell viability, surface markers, and multiple differentiation characteristics. AS BM-MSCs induced an imbalance in the ratio of CCR4^+^CCR6^+^ Th/Treg cells by reducing Treg and increasing CCR4^+^CCR6^+^ Th cells. AS MSCs reduced Foxp3^+^ cells when co-cultured with PBMCs.	[123]
Systemic lupus erythematosus	Immunocytochemistry and flow cytometry with CD34, CD45, CD73, CD90, CD105, CD31, CD19, CD11b, HLA-ABC, CD44, CD29, and HLA-DR surface markers. qPCR with IL6, IL8, Gro1, Mcp2, Rantes, and GM-CSF. Western blotting for FNβ, MAVS, p53, p16, and 53BP1, ELISA for IL-6, IL-8, and GM-CSF. Comet assay. β-galactosidase assay.	SLE BM-MSCs were characterised by: ○ reduced proliferation rate. ○ increased production of reactive oxygen species. ○ increased expression of p53 and p16. ○ altered cytokine production, increased IL6 and IL8, increased IFNβ levels, and IFNβ-induced mRNAs.	[127]
Systemic sclerosis	Quantification of CFU-F. Osteogenic, adipogenic, and endothelial cells differentiation. Immunophenotyping by flow cytometry. Assessment of the endothelial-like MSC (EL-MSC) phenotype after culture in endothelial-specific medium and measurement of VEGFR and CXCR4 expression with flow cytometric analysis. Chemoinvasion assays of MSCs and EL-MSCs. Capillary morphogenesis assay. Telomerase activity assay.	SSc MSCs demonstrated: ○ The same phenotype (positive for CD29, CD44, CD166, CD90, CD73, HLA–A, B, and C, and CD105, low HLA–DP, DQ, and DR) and clonogenic activity as healthy MSCs. ○ A decreased percentage of VEGFR-2^+^, CXCR4^+^, VEGFR-2^+^/CXCR4^+^, and early senescence. ○ Low migration and angiogenic potential. ○ Decreased capacity to capillary morphogenesis and chemoinvasion. ○ The addition of VEGF and stromal cell-derived factor 1 to cultured SSc EL-MSCs increased their angiogenic potential less than that in controls.	[124]
Parkinson’s disease	Confocal images for identification of mitochondrial and lysosomal localisation. NADH autofluorescence. Nuclear DNA sequencing analysis with target genes: SNCA, PARK2, UCHL1, PINK1, DJ1, LRRK2, GBA, VPS35, ATP13A2, EIF4G1, HTRA2, DNAJC13, VPS13C, DNAJC6, FBXO7, PLA2G6, SYNJ1, and MAPT. Mitochondrial DNA sequencing analysis. MSC adipogenic potential.	Impaired differentiation of BM-MSCs. Mitochondrial dysfunction. Higher basal rate of mitochondrial degradation and lower levels of biogenesis. Reduction in mitochondrial mass. Increased level of oxidative stress.	[121]
Idiopathic pulmonary fibrosis	Cell senescence was determined by cell proliferation and expression of p16^INK4A^, p21, and β-galactosidase activity. Mitochondrial function and DNA damage were measured. Paracrine induction of senescence and profibrotic responses were analysed in human lung fibroblasts. The reparative capacity of BM-MSCs was examined in vivo using the bleomycin-induced lung fibrosis model.	BM-MSCs from patients with IPF characterised by: ○ Mitochondrial dysfunction. ○ Accumulation of DNA damage. ○ Diminished migration capacity of MSCs. ○ Less effectiveness in preventing fibrotic changes in mice after bleomycin-induced injury, increasing illness severity, and pro-inflammatory responses.	[120]
Chronic obstructive pulmonary disease	Immunophenotyping of MSCs by flow cytometry using CD73, CDw90, CD105, CD45, CD14, and CD34. Tri-lineage differentiation. The expression of migration-related chemokine receptors and their ligands in BM-MSCs: qPCR with SDF-1a, CXCR4, CCR7, CCL19, and CCL21. SDF-1α levels in MSC-conditioned media and sera evaluated by ELISA.	COPD BM-MSCs were positive for CD73, CD90, and CD105 and negative for CD45, CD14, and CD34 antigens, and were capable of differentiating towards the adipogenic, osteogenic, and chondrogenic lineages. CXCR4 mRNA expression were decreased in COPD BM-MSCs that provided the evidence that CXCR4/SDF1 is dysregulated in COPD patients. COPD affects SDF1α levels in serum and BM-MSCs.	[125]

**Table 5:** Advantages and disadvantages of autologous versus allogeneic MSCs. The pros and cons of autologous and allogeneic MSCs transplantation were summarised in the prospect of cell availability, quantity, and quality.

	Allogeneic	Autologous
Availability	Immediate “off-the-shelf” availability	Need to be taken, isolated, and cultured
Quality	Control of donor age (may be selectively derived from young) Cells from healthy donors	No control of donor age Potential disease impairment of MSCs
Cell quality in accordance with good manufacturing practice	Screening for chromosomal aberrations, viral contamination, sterility, identity, purity, and cell potency	Usually, no screening for cell potency due to lack of time and material
Quantity	Standardising the quantity of cells	Difficulties to grow in culture and yield low cell numbers
Immune response on MSCs transplantation	Can be recognised by immune response and rejected	Are not recognised by immunocompetent cells because of the usage of their own cells with the same antigens

## Data Availability

Not applicable.
